# Exploring past and future fluency of temporal landmarks under reduced agency

**DOI:** 10.1038/s41598-025-00530-4

**Published:** 2025-05-07

**Authors:** Alexandra Zimbatu, Steven J. Bickley, Stephen Whyte

**Affiliations:** 1 School of Advertising, Marketing and Public Relations, Queensland University of Technology, Brisbane, 4000 Australia; 2 Department of Marketing and Supply Chain Management, School of Business and Economics, Maastricht University, 6211 Maastricht, Limburg The Netherlands; 3 School of Economics and Finance, Queensland University of Technology, Brisbane, 4000 Australia; 4 Australian Research Council Centre for Behavioural Insights for Technology Adoption, Queensland University of Technology, Brisbane, QLD 4000 Australia; 5 Australian Research Council Centre for Cell and Tissue Engineering Technologies, Queensland University of Technology, Brisbane, QLD 4000 Australia

**Keywords:** Temporal landmarks, Wellbeing, Past and future fluency, Time work, Anticipation, Quality of life, Health services

## Abstract

Significant temporal landmarks often involve high emotionality, thereby deeply imprinting within one’s autobiographical memory. In this study we explore the connection between anticipation, temporal landmarks, subjective passage of time and perceived wellbeing. We do this by conducting a three-stage mixed method study including qualitative thematic analysis, AI-assisted coding and analysis, and quantitative negative binomial multivariate analysis of the Past Fluency and Future Fluency reported events (*n* = 73,244) provided by *n* = 1,113 participants across the six reported (future and past) temporal horizons (One Week, One Month, One Year) in the *Blursday Database* during the COVID-19 pandemic. Our findings show that participants state more past Temporal Landmarks compared with future, while the opposite is true for Utilitarian Activities. Key sex differences exist in men’s vs. women’s identification of Utilitarian Activities (greater future fluency), Temporal Landmarks (greater past fluency) and fewer Discretionary Activities listed for both past and future fluency. Statistically significant relationships are found with responses about stringency of lockdown restrictions and subjective assessments of confinement. Our study provides novel methodology and findings that demonstrate how engagement in anticipatory behaviour can be considered a type of *time work*, where an individual intentionally alters or customizes temporal experiences during times of reduced agency.

**Supplementary Information:**

The online version contains supplementary material available at 10.1038/s41598-025-00530-4.

## Introduction

Time-based orientation points serve an important sense-making function, supporting individuals in structuring their ‘life narrative’ into mental accounting periods, and organizing their understanding of the world^1^. Larger scale temporal events are known as Temporal Landmarks, describing events that stand out in an individual’s perception as being distinct from the mundanity and routine of life^1,2^. Often times, these Temporal Landmarks are culturally salient and acknowledged (i.e. Easter, New Year, Labor Day), or reflect traditionally celebrated social milestones related to normative progression through the life course (i.e. graduations, weddings, anniversaries or child rearing)^3^. Having larger-scale Temporal Landmarks has been shown to regulate mortality-related sadness and support bigger picture thinking^4^, as well as being used by individuals as anchors to motivate aspirational and goal-oriented behaviour^5^.

On a smaller scale, micro-level temporal events, such as looking forward to a monthly dinner with friends, can also be equally powerful in generating utility for individuals in the present – suggesting that the anticipation of Temporal Landmarks is an autotelic process^6^. Interestingly, in circumstances of low controllability (such as the COVID-19 pandemic), indulging in a positive fantasy (i.e. anticipation) of a future temporal event can be a beneficial self-regulation strategy^7,8^. This adaptive role of anticipation and time structuring as a way to manipulate subjective perceptions of time, known as ‘*Time Work’*, requires further scientific exploration, being a potentially notable and accessible way to exercise control and agency^9^. Given the current climate of growing uncertainty worldwide, identifying how individuals exercise agency and control in situations of low controllability can provide important insights for behavioural science as a whole. Specifically, identifying *what kind* of temporal events should be structured and anticipated to generate utility, and *how frequently* they should be structured to maximise benefit, is currently unknown. Addressing this research gap is an important step in uncovering the individual behaviours that support us in maintaining our sense of control and wellbeing in settings of reduced agency.

Indeed, theoretical and empirical work from behavioural sciences provides evidence into the important role temporal events and landmarks play in an individual’s utility. This is particularly valuable for the exploration of human behaviour under regulation or constraint across a range of applied settings. For instance, higher education and university settings use a variety of temporal landmark *types* (e.g. utilitarian and functional landmarks like assessments and exams) to track student progress, and social, recreational landmarks to keep students engaged (e.g. orientation week parties, mid-semester break, graduation ceremonies). These temporal events are used in various combinations and made salient to students as mechanisms to address attrition, but to also create a sense of progress and temporal orientation during the extended and long-term time frame of a multi-year degree^10^. Another example is in corrective services and prison settings, whereby policy makers use the temporal landmark of ‘visitation hours’ as a tool to reduce misconduct and rule infractions^11^. In the business and commerce domains, temporal manipulation is well documented and practiced in managing the waiting experience – for example, food and beverage services in domestic and internation airline travel, or well-designed progress points during long queues at Disneyland^12^.

While these mechanisms to manipulate subjective perceptions of time are examples created by the service provider *for* the consumer due to power asymmetry, understanding *individual efforts* to structure temporal orientation points is arguably even more important. The COVID-19 pandemic provides a unique opportunity to explore human behaviour during a collective reduction in agency^7,13–15^, with notable existing work already connecting wellbeing and time perception during COVID-19^16–18^. We extend this knowledge by conducting a novel descriptive analysis of temporal orientation points created and used by individuals to generate utility during their own extended periods of ‘empty time’. Using a three-stage mixed method, we explore the nature of self-reported past and future temporal activities and landmarks on individual utility, and outline the implications for individuals, businesses and policy-makers alike.

## Background

Individuals strategically think about events in a way that benefits the self or serves a protective function, with Temporal Landmarks altering one’s perceived connection between present and future identities^2^. In situations where one is unable to anchor to or envisage salient future-oriented events, there can be adverse effects on happiness, health, wellbeing and even broader life satisfaction^19^. Further, recent work suggests that having limited focal events to direct positive anticipation towards – particularly ones that have emotional vividness or significance to support memory encoding – impacts one’s subjective perceptions of time^20^. This is particularly the case in contexts of confinement, whereby individuals have long periods of unstructured time. This ‘empty time’ is either voluntarily filled, left empty, or structured on behalf of the person with diminished agency (e.g., the prison context)^21^. For many without external stimuli coming in, structuring meals is used as a key temporal orientation point that attempts to add structure to the passage of time^22^ and acts as a ‘time filler’ during situations like the pandemic^23^. With no distinctive temporal landmark on the horizon, the blending of the chapters in one’s life also creates limited formal or ‘salient’ identifiable opportunities for an individual to wipe their ‘slate clean’, start fresh or break a pattern of behaviour^24^. Individual efforts and behavioural practices directed towards manipulating one’s perception of time in order to generate utility is known as ‘time work’^9^. Under this conceptualization, an individual directs their agency towards modifying their temporal experience through a number of mechanisms, such as customising the *sequence* of activities, *frequency* of a behaviour or activity, *timing* (when something happens), *duration* or *allocation* (‘setting time aside’ for tasks that reflect values). These cumulatively reflect practices that disentangle objective time (i.e. clock time), from ‘felt time’^9^. Engaging in time work is an important source of efficacy and personal satisfaction, providing an opportunity to exercise control in a context where agency is reduced^9^.

Notable work^16–18^ has been undertaken to explore wellbeing, time perception and human behaviour during the shared experience of reduced agency during the pandemic. The element of agency and perceived control during the pandemic is in and of itself a novel area for researchers and policy makers alike. Research undertaken during the pandemic highlights that mental time travel to notable individual or collective (positive) temporal landmarks has important links to agency and perceived control. Specifically, people attribute more agency to themselves in relation to positive rather than negative events in times of reduced agency^15^. We extend these interesting insights by exploring *what kind of* temporal landmarks are used by individuals in times of reduced agency, and how frequently they are used. While we know that there are notable types of behaviours related to customising one’s subjective experiences of time^9^, to date, there have been limited attempts to summarise what any of these key temporal orientation points actually are. Further, little is known regarding the frequency by which individuals actively create or use these landmarks to add structure to their own perceived passage of time. We utilize a unique collection of self-reported data (*n* = 73,244) as our dependent variable of interest, captured from *n* = 1,113 participants survey responses as part of the *Blursday Database*^16^. To examine the descriptive nature of these milestones, activities and temporal landmarks that individuals use as foci of anticipation and anchors to structure time’s passage, we conduct a three-stage mixed method approach. This consists of a qualitative thematic analysis, an AI-assisted coding and analysis process, and a quantitative multivariate analysis of the more than seventy thousand “Past Fluency” and “Future Fluency” reported landmark events provided by the *n* = 1,133 participants from the *Blursday Database*. We analyze the composition of these self-reported milestones, as well as their frequency/volume, and any relationship or impact on perceived wellbeing in the sample populations. We categorise the reported results into four main themes, distinguishing between participant responses related to:

*Temporal landmarks*—Broadly speaking, a temporal landmark can be defined as any event that is distinct to the mundanity of everyday life. Further analysis can be used to distinguish between *calendar* temporal landmarks (i.e. Easter, New Year, Memorial Day), personal narrative events (i.e. moving out of home, relationship break up, promotion, moving overseas), personal temporal landmarks (birthdays, anniversaries), reference points and facts of life experiences (i.e. illness, bereavement)^1,2,25^.

*Discretionary activities*—Relates to voluntary activities that are used as modes of filling one’s free or empty time. Often voluntarily chosen and structures as a way to find pleasure, relaxation or fun, support personal growth, cope with stress or as part of identity affirmation^26^. For instance, social events, family gatherings, travel, entertainment and recreational activities.

*Utilitarian activities*—Utilitarian activities relate to any of the events and actions that can often not be skipped. These include personal care activities (i.e. fulfilling basic needs), domestic activities relating to upkeep, and even paid activities such as school or employment related activities^27^.

*Evaluation*—Rather than a specific activity, an evaluation refers to an objective evaluation of one’s circumstances. This theme encompasses participant responses simply providing a positive evaluation (e.g. *things are good*), negative evaluation (e.g. *I’m sad*) or specifically referencing lockdown restrictions (e.g. *staying home*, *can’t go anywhere*).

Thus, in our analysis of over seventy thousand landmark events reported by participants (i.e. our dependent variable) we examine how varying compositions of these landmarks (e.g., discretionary versus utilitarian activities, with others or alone, passive or active) impact subjective perceptions of confinement, time perception and wellbeing (i.e., felt/reported loneliness).

## Method and materials

### Data

We draw upon data collected via the Blursday Project, a multicentric set of tasks and questionnaires designed specifically to assess time perception during COVID-19 across twelve countries (Italy, United Kingdom, Canada, France, Germany, Argentina, Greece, Turkey, Colombia, India, Japan, United States). These data and details about the collection process are freely available under the Non-commercial, Research-only licensing from Gorilla Open Materials Attribution (https://app.gorilla.sc/openmaterials/278377).

To understand temporal landmarks, time work and anticipation, we were specifically interested in participant’s open-text responses collected via two tasks captured in the Blursday Project’s repository being: *Future Fluency* and *Past Fluency*. In the Future Fluency task, an instrument initially developed by Macleod et al. (1997)^28^ and adapted for the Blursday study (seen under ‘Future Fluency’ here:https://app.gorilla.sc/openmaterials/278377 participants were prompted to report as many individual events as possible (i.e., in one minute per question/time horizon) that they expect or look forward to occurring across three future time horizons (i.e. in one week, one month and in one year). Similarly, also under a time limit of one minute per question/horizon, the Past Fluency task (seen under the ‘Past Fluency’ task here: https://app.gorilla.sc/openmaterials/278377) asked participants to report as many individual events that already occurred across three time horizons (i.e., last week, last month and last year). This is the primary (unstructured) qualitative data in this study.

Building on the original Blursday dataset, the *Stringency-Confinement Difference* metric was constructed to compare objective and subjective measures of confinement during the pandemic. First, we normalized the *Stringency Index* (ranging from 0 to 100) and *Subjective Confinement* (ranging from 5 to 20) to ratios between 0 and 1. Next, we calculated the difference between the normalized *Stringency Index* (a measure of objectively imposed restrictions) and the normalized *Subjective Confinement* (a self-reported proxy for perceived isolation).

### Ethics statement

The ethics approval process for original data collection was approved by University Paris-Saclay (CER-Paris-Saclay-2020-020) and described in detail in the original Blursday publication^16^. All study participants provided informed consent for data collection and data sharing. These data and details about the collection process are freely available under the Non-commercial, Research-only licensing from Gorilla Open Materials Attribution (https://app.gorilla.sc/openmaterials/278377). All data for the current study was de-identified, and both described and analysed at the aggregate level.

### Qualitative thematic analysis

Noting that the intention of this research was to identify the *type of* temporal events used by participants, the basis of the data included in the analysis is qualitative. Participant responses to Future Fluency and Past Fluency tasks were in sentence format, or via short strings of text. As such, thematic analysis was necessary to appropriately categorise and systematically group the content of the temporal events across such a large scale. Following recommended best practice in qualitative analysis^29^, a codebook was developed to facilitate the grouping and consistent, repeatable thematic analysis of participants’ descriptive responses.

The codebook involved articulating both open and axial codes (see^30^ for detailed descriptions). This means that the first order, literal descriptions (e.g. participant response of ‘vacuuming’) would connect to a higher order category (e.g. response of ‘vacuuming’, would form part of ‘Household Obligations’). There are of course many categories that may emerge from the dataset, that again need to be grouped into logical themes based on commonalities in order to consolidate meaning (i.e. ‘Household Obligations’ is one of the categories within the ‘Utilitarian Activities’ theme)^31^. While each response was coded at both the category and theme level, our analysis here reports the overarching Themes that were prevalent (i.e. Temporal Landmarks, Discretionary Activities, Utilitarian Activities, Evaluations). Development of the codebook was iterative and abductive in nature, initially led by existing literature on temporal landmarks (i.e.^1,2^, and discretionary and utilitarian activities (i.e. Activities of Daily living –^32^). An extract of the coding book is provided in Table 1 below, populated with real strings of from our qualitative data collected:

**Table 1:** Extract from the coding book.

Utilitarian activities		
Relates to functional activities that can often not be skipped. These include personal care activities (i.e. fulfilling basic needs), domestic activities relating to upkeep, and even paid activities such as employment-related activities		

Code	Description	Example/Keywords
Household obligations	Activities related to maintaining one’s home and other basic needs	Cleaning; maintenance Grocery shopping; yard work
Physiological needs and personal care	References made to the fulfillment of one’s foundational, core physiological needs (eating, sleeping), alongside personal care activities	Sleep; Ordering food; Meal Preparation; Cooking; Eating; Showering
Work/School activities	Attending to core activities related to one’s paid employment or study	Meetings; Training session; Assignments; Attending lecture

Coding was performed by three independent research assistants on a small subsection of the entire dataset (i.e., focusing on the Past Fluency and Future Fluency responses from only the UK, US, and Canada). Interrater consistency checks were performed, meaning that each coder conducted their separate coding of a small subsection of the results, before coming together with the broader research team to compare results^33^. Any coding discrepancies, often arising due to interpretation and experience levels of the coders, were discussed until a consensus was achieved. Any new codes emerging from the data were discussed amongst the authors of the study and iterated into the coding book^30^. This approach is commonly employed within qualitative research, emphasizing interrater consistency checks rather than only (quantitative) scores of interrater reliability when dealing with emergent constructs^33,34^.

As a robustness check, to quantitatively assess interrater reliability resultant from the qualitative process followed above, we computed both Fleiss’ Kappa and Cohen’s Kappa values, as well as overall agreement percentages, across four top-level categories (Discretionary Activities, Evaluations, Temporal Landmarks, and Utilitarian Activities). For example, in the Discretionary Activities category, the “Humans Only” subset achieved a Fleiss’ Kappa of 0.9218 with Cohen’s Kappa values of 0.9691, 0.9461, and 0.8851, yielding an overall agreement of 92.57%. In the Evaluations category, the “Humans Only” subset showed a Fleiss’ Kappa of 0.7555 (with Cohen’s Kappa values ranging from 0.5602 to 0.8533) and a 97.77% agreement (see Table S2 in the Supplementary Information for detail). This robust combination of qualitative consensus-building and quantitative reliability checks confirms the credibility and consistency of our coding approach.

### AI-assisted coding and analysis process

To enhance the efficiency and productivity of the coding over 73,244 responses from 1,133 participants, we employed OpenAI’s APIs to scale our thematic analysis. APIs (Application Programming Interfaces) are tools that allow software to communicate with external services—in this case, enabling us to interact programmatically with OpenAI’s models to automate and scale our qualitative analysis. Specifically, we interacted with the assistants API during the initial data collection period (April to May 2024) and later employed the chat completions API in structured outputs mode (available from August 2024) during the second data collection period (November 2024), with the gpt-3.5-turbo-0125 and gpt-4o-2024-08-06 models, respectively. We kept the model’s temperature within ChatGPT’s default range (0.7–0.8) for more creative/qualitative tasks, while all other parameters (top_p, frequency_penalty, presence_penalty, logit_bias, etc.) remained at their default API settings.

During the first round of data collection (April–May 2024), we relied on the assistants API in combination with extensive regex expressions to extract and code the data, because at the time of implementation, there were no standardised/json output modes available. In the second round (November 2024), we leveraged the structured outputs mode of the chat completions model to standardize and clean the data collected in the first round. This second round also allowed us to address any missing observations by re-running the coding process using the updated model.

Our approach draws on recent studies that demonstrate two complementary strategies for integrating LLMs with human coding. Recent research^35,36^ outlines inductive coding methods in which LLMs are allowed to generate new code suggestions alongside human annotations; for example^35^, integrated LLMs with human coders by permitting the model to propose alternative or additional codes, thereby enriching the codebook and improving inter-annotator agreement. In contrast^37^, focused on applying LLMs to deductive coding by comparing the LLM’s performance to human coders across established coding tasks. For example, one task the coding of news articles in the BBC News dataset, where each article had to be classified into one of five mutually exclusive topics (technology, business, politics, entertainment, or sports), and the model’s outputs were directly compared against a fixed, expert-defined codebook. The study reported that the LLM achieved an inter-rater reliability—using metrics like Gwet’s AC1—that was approximately 0.80, indicating a high level of agreement with human coders. In our work, we adopted a more inductive strategy in phase 1—permitting the LLM to generate new suggestions for the codebook within the system message/prompt—while in phase 2 we aligned more with the deductive approach by restricting the LLM’s responses to a predefined JSON schema corresponding to the final codebook and removing the explicit permission in the system message/prompt from phase 1. In contrast^37^, focused on these established deductive coding tasks without employing such a structural constraint on the output.

Given the multilingual nature of the Blursday dataset, which includes responses from participants across twelve countries (Italy, United Kingdom, Canada, France, Germany, Argentina, Greece, Turkey, Colombia, India, Japan, and the United States), we translated non-English responses into English using Google Cloud Translation APIs via the googleLanguageR package. We mapped the Task_Name field from the Blursday dataset to the corresponding language rather than relying on autodetection of the source language to minimize likelihood of translation errors. Finally, while the initial language translation involved a mix of Google Cloud tools and R code (version 4.3.1), the remainder of the workflow—including all interactions with the OpenAI models/APIs, producing figures, conducting regressions, and performing statistical analyses—was implemented in Python (version 3.11.0).

Importantly, we confirmed that the interrater consistency between the large language model outputs and human coders was statistically similar. For instance, comparable reliability was found when comparing the coding of the so-called “expert RA” coder with the AI, as well as between the two “novice RA” coders and the AI—with Fleiss’ Kappa values ranging from 0.75 to 0.92 and agreement percentages between 82.16% and 99.15% (see Table S2 in the Supplementary Information for details), ensuring reliability before we scaled up to the full dataset.

### Quantitative multivariate analysis

We conducted separate regression analyses using the mean and frequency of participants’ Past and Future fluency responses as our dependent variables of interest. Our independent variables of interest included socio-demographic data on individual differences such as age and sex. Additionally, we incorporated control variables related to participants’ self-reported perceptions of loneliness, specifically Felt Loneliness and Reported Loneliness. We also measured aspects of reduced agency, both actual and perceived, using the Stringency Index, Subjective Confinement, Confinement Duration, and derived Stringency-Confinement Difference measurement constructs. As our key (dependent) variable of interest is a count variable, and as participant responses are not necessarily related, we perform negative binomial and Poisson regression analysis, providing also marginal effects results for interpretation of coefficients.

### Methodological limitations

Given our approach outlined above, certain technical and methodological limitations warrant consideration. First, our dataset spans twelve countries, with responses originally provided in multiple languages. Although we mitigated potential translation errors by mapping responses to the corresponding source language for the Google Cloud Translation API—rather than relying on the auto-detect feature—subtle nuances in meaning may have been lost. Such nuances could influence thematic interpretation by both the human research assistants (Australian postgraduate research students) and the AI/LLM coders, who may exhibit biases (e.g., more Western/US-leaning^38^ or aligned with rational, chain-of-thought reasoning) due to OpenAI’s training data and/or AI safety-trained protocols.

Second, our qualitative thematic analysis followed an iterative inductive consensus-building process for developing the codebook, wherein emergent constructs were identified and refined through discussion among the research team. This approach allowed our team to generate new code suggestions alongside preexisting categories, thereby enriching the codebook, but is also inherently subjective despite our robust interrater reliability metrics (with Fleiss’ Kappa values ranging from 0.75 to 0.92, see Table S2 in Supplementary Information). Therefore, while this process was essential for capturing emergent constructs, some degree of interpretative variability remains, potentially influencing the coding outcomes. Moreover, the integration of AI-assisted coding with human coding, which significantly enhanced efficiency in processing over 73,000 responses, introduces its own limitations. For instance, AI models (e.g., LLMs like gpt-3.5-turbo and gpt-4o) can exhibit significant biases, such as reinforcing gender stereotypes and racial misclassifications^39^, and the models’ ideological leanings reflecting their creators’ socio-political contexts^40^. Likewise^41^, show the same model’s reported accuracy can vary by up to 15–20% depending on which benchmark is used for evaluation, highlighting how different datasets or testing conditions can influence performance metrics, thereby introducing an additional potentially subjective element.

Third, our quantitative analyses—employing negative binomial and Poisson regression models on count data derived from self-reported measures—also face notable challenges. For instance, measurement errors may be present as self-reported counts are inherently prone to inaccuracies due to recall bias or social desirability effects—for example, participants might inadvertently overestimate or underestimate their recalled events, especially under the time constraints (1 min) of the Past and Future Fluency tasks. Additionally, potential confounding factors such as unmeasured socio-demographic variables (e.g., education level, socio-economic status) or cultural differences in interpreting what constitutes a significant event may influence both the reporting of events and perceptions of confinement. The constructed Stringency-Confinement Difference metric, while informative, also simplifies the complex, multidimensional experience of confinement by assuming a linear relationship between objective government stringency measures and subjective confinement perceptions. Finally, given that the original Blursday data were collected exclusively during the COVID-19 pandemic and within a specific set of countries, these factors may limit the broader generalizability of our findings to other contexts and or time periods.

### Descriptive statistics

In Table 2 we provide the demographic breakdown of our sample population taken from the Blursday Database^16^ including participants from Italy, United Kingdom, Canada, France, Germany, Argentina, Greece, Turkey, Colombia, India, Japan, and United States. In Table 3 we provide percentages and total counts of the temporal events reported by participants (i.e. our dependent variable of interest), across the three temporal projections relating to Past Fluency and Future Fluency (week, month, year). In Table 4 we provide total counts and percentages across our four primary-level activity categories; Utilitarian, Discretionary, Landmark, Evaluation, and Other.

**Table 2:** Demographics of the sample population drawn from the *Blursday* dataset.

Variable	Category	Count	Percentage	Mean	Std	Min	Max
Sex	F	42,832	64.9	-	-	-	-
	M	23,141	35.1	-	-	-	-
Country	JP	20,838	30.2	-	-	-	-
	FR	14,473	21	-	-	-	-
	IT	12,323	17.8	-	-	-	-
	TR	7534	10.9	-	-	-	-
	GR	3985	5.8	-	-	-	-
	DE	3882	5.6	-	-	-	-
	AR	3567	5.2	-	-	-	-
	IN	1189	1.7	-	-	-	-
	CA	510	0.7	-	-	-	-
	US	300	0.4	-	-	-	-
	UK	261	0.4	-	-	-	-
	CO	213	0.3	-	-	-	-
Age	-	65,973	-	35.14	14.37	18.4	80.2
Stringency index	-	69,576	-	62.26	22.05	25.93	96.3
Reported loneliness	-	66,977	-	5.50	1.62	2	8
Felt loneliness	-	66,977	-	9.08	2.11	3	12
Subjective confinement	-	66,977	-	14.58	3.36	5	20
ConfDuration	-	22,696	-	79.13	77.82	2.45	1110.34

**Table 3:** Percentages and total counts of Temporal events reported across the future fluency and past fluency tasks for the three Temporal projections (week, month, year).

Task name	Screen number*	Count	Percentage (%)
Future fluency	1	13,753	18.8
	2	12,501	17.1
	3	12,070	16.5
Past fluency	1	12,233	16.7
	2	11,184	15.3
	3	11,503	15.7

*Screen number 1 (week), 2 (month), and 3 (year), ask respondents to “Please, write as many events as possible that [have occurred/could occur] in the [last/coming] [week/month/year]”, respectively.

**Table 4:** Total counts and percentages across the primary-level activity categories.

Primary-level category	Count	Percentage (%)
Utilitarian	27,613	39.51
Discretionary	29,143	41.70
Landmark	14,071	20.14
Evaluation	5730	8.20
Other	762	1.09

## Analysis

**Fig. 1:**
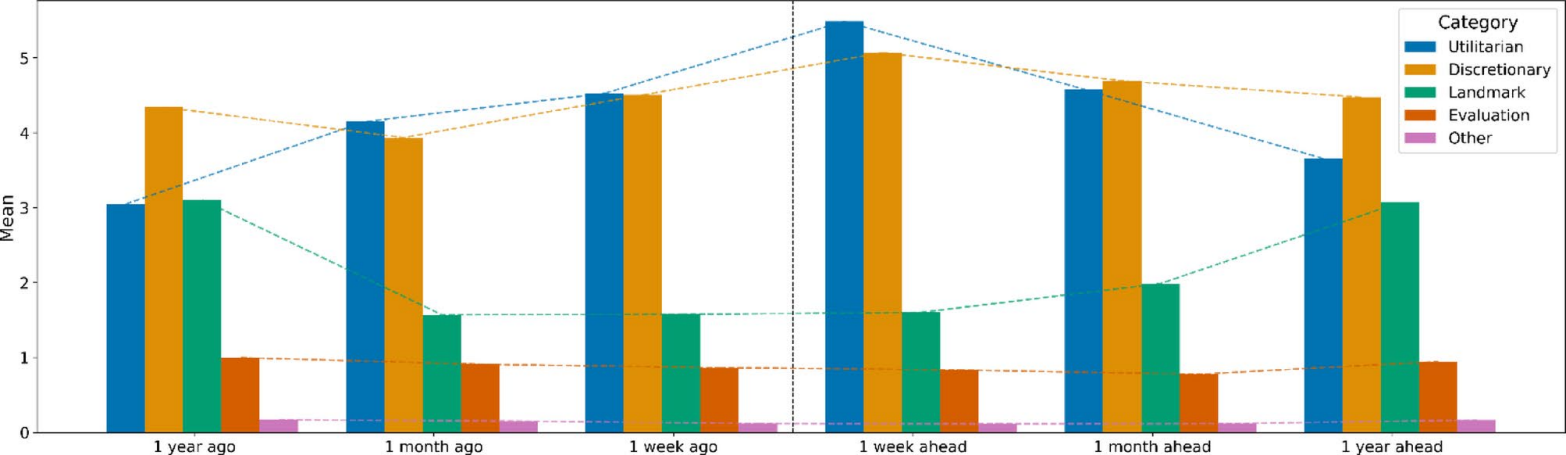
Grouped bar plot with mean counts per person at primary category level. *Note*: See Supplementary Materials for grouped bar plots with frequency counts per person at primary category level, and detailed breakdowns per coding category.

We begin our analysis with a visualised exploration of frequency and average participant responses across the six past and future timeframes, differentiated by our four primary coding categories. We find that mean-differences for our total count categories (Fig. 1, also see Table 5) show that three categories had highly statistically significant differences between total past and future fluency counts; Temporal Landmarks, Utilitarian Activity, and the Evaluations category, all at a 1% level (*p* = < 0.01).

**Table 5:** Comparative t-test results for combined average number of responses for future vs. past by four primary categories.

Category: Primary count	Future mean	Past mean	Mean difference	T-statistic	*P*-value	Significance
Temporal landmarks	0.159	0.171	-0.012	-4.072	0	***
Utilitarian activities	0.296	0.277	0.019	5.406	0	***
Discretionary activities	0.44	0.433	0.007	1.637	0.1016	
Evaluations	0.045	0.057	-0.013	-7.676	0	***

*** = *p* < 0.01, ** = *p* < 0.05, * = *p* < 0.1.

When we explore direct comparisons for past and future fluency (e.g. comparing mean of utilitarian responses for one month in the future vs. one month in the past) we find statistically significant results for both week (*p* = < 0.01) and year (*p* = 0.0134) for Temporal Landmarks with a negative mean difference. That is, past Temporal Landmarks are more frequently recalled compared to future Temporal Landmarks. The opposite is true for Utilitarian Activities with statistically significant results for both week (*p* = < 0.01) and year (*p* = < 0.01) in which participants recall greater future events as opposed to past events. See Table 6. For Discretionary Activities we see participants recalling more items for one month in advance, compared to one month in the past (*p* = < 0.01). And for the Evaluations category, participants recall more past events (relative to future events) in all three time categories of week, month and year (*p* = < 0.01), see Table 5.

**Table 6:** Comparative t-test results for future vs past differences by time category.

Category	Comparison: Future vs. past	Future mean	Past mean	Mean difference	T-statistic	*P*-value	Significance
Temporal landmarks	Week	0.109	0.13	-0.021	-4.8	0	***
	Month	0.143	0.138	0.005	1.117	0.264	
	Year	0.234	0.249	-0.015	-2.474	0.0134	**
Utilitarian activities	Week	0.34	0.314	0.026	4.261	0	***
	Month	0.299	0.305	-0.006	-0.949	0.3427	
	Year	0.242	0.21	0.032	5.604	0	***
Discretionary activities	Week	0.43	0.429	0	0.051	0.959	
	Month	0.45	0.416	0.035	4.443	0	***
	Year	0.441	0.453	-0.012	-1.485	0.1375	
Evaluations	Week	0.045	0.053	-0.008	-3.003	0.0027	***
	Month	0.043	0.062	-0.02	-6.734	0	***
	Year	0.046	0.057	-0.01	-3.568	0.0004	***

*** = *p* < 0.01, ** = *p* < 0.05, * = *p* < 0.1.

In Table 7 we present our negative binomial multivariate results for each the categories, of Discretionary Activities, Utilitarian Activities, and Temporal Landmarks with an individual specification for both past fluency and future fluency (we present the same model specifications with the addition of interaction effects with our sex dummy in Table S4 of the *Supplementary Materials*). We begin with the results for age and sex, where we see that for age, the older the study participant, the more past Temporal Landmarks they recall as part of the task – consistent with research suggesting that older adults have enhanced recall on semantic components of autobiographical memory^42^. We find several highly statistically significant sex differences, with men (compared to women) recalling less discretionary activities in both the past and future. Further, we also observe that men recall more future Utilitarian Activities and more past Temporal Landmarks when compared to their female counterparts.

For the frequency that participants provide and any relationship with felt loneliness and reported loneliness, we only find a relationship with recall of past Temporal Landmarks. That is, participants who experience higher felt loneliness report greater numbers of past Temporal Landmarks in their recall task.

As one of this study’s core focuses is relating to perceptions of time under constrained agency during COVID-19, it is not surprising that our regression analysis finds statistically significant results for the Stringency Index independent variable in five of our six specifications. Participants experiencing higher stringency measures recalled less past Utilitarian Activities. Participants experiencing higher stringency measures also recalled more past and future Discretionary Activities, and more past Temporal Landmarks. Further, those who had higher Subjective Confinement scores (i.e. slower felt speed/passage of subjective time) reported more Temporal Landmarks in both the past and future fluency tasks. Finally, for actual Confinement Duration, we see that participants who have spent longer periods of time under reduced agency report more future Utilitarian Activities compared with those who have endured less time in confinement.

**Table 7:** Regression results (Negative Binomial) across the past/future fluency tasks with demographics and other control variables relating to COVID-19 measurement proxies.

Independent variables	Utilitarian activity		Discretionary activity		Temporal landmark	
	Past fluency	Future fluency	Past fluency	Future fluency	Past fluency	Future fluency
Age	-0.001 (-0.703) *-0.000*	-0.002 (-1.384) *-0.001*	0.000 (0.187) *0.000*	0.002 (1.300) *0.001*	0.005** (2.441) *0.001*	0.001 (0.334) *0.000*
Sex (Male dummy)	0.051 (1.154) *0.014*	0.122*** (2.926) *0.036*	-0.147*** (-3.791) *-0.064*	-0.084** (-2.322) *-0.039*	0.146*** (2.671) *0.023*	0.028 (0.487) *0.004*
Felt loneliness	0.023* (1.962) *0.006*	0.020* (1.827) *0.006*	0.010 (1.035) *0.004*	0.001 (0.074) *0.000*	-0.028* (-1.942) *-0.004*	0.007 (0.469) *0.001*
Reported loneliness	-0.019 (-1.503) *-0.005*	-0.022* (-1.791) *-0.006*	-0.015 (-1.351) *-0.006*	-0.003 (-0.244) *-0.001*	0.044*** (2.698) *0.007*	0.014 (0.871) *0.002*
Stringency index	-0.004*** (-3.675) *-0.001*	-0.000 (-0.394) *-0.000*	0.003*** (3.099) *0.001*	0.004*** (5.063) *0.002*	0.004*** (2.603) *0.001*	-0.002* (-1.676) *-0.000*
Subjective confinement	0.004 (0.805) *0.001*	-0.002 (-0.389) *-0.000*	-0.005 (-1.222) *-0.002*	-0.002 (-0.531) *-0.001*	0.015*** (2.770) *0.002*	0.022*** (3.855) *0.003*
Confinement duration	-0.000* (-1.667) *-0.000*	0.001** (2.151) *0.000*	0.000 (1.236) *0.000*	0.000 (1.636) *0.000*	0.000* (1.696) *0.000*	-0.001 (-1.383) *-0.000*
Screen 2 (One month)	0.028 (0.584) *0.008*	-0.145*** (-3.223) *-0.042*	-0.033 (-0.757) *-0.014*	0.045 (1.130) *0.021*	-0.050 (-0.716) *-0.008*	0.334*** (4.771) *0.045*
Screen 3 (One year)	-0.371*** (-7.241) *-0.102*	-0.365*** (-7.743) *-0.107*	0.102** (2.454) *0.044*	0.098** (2.455) *0.045*	0.603*** (10.182) *0.094*	0.776*** (12.391) *0.104*
Intercept	-0.990*** (0.000) *0.000*	-1.090*** (0.000) *0.000*	-1.002*** (0.000) *0.000*	-1.170*** (0.000) *0.000*	-2.756*** (0.000) *0.000*	-2.641*** (0.000) *0.000*
*N*	10,412	11,323	10,412	11,323	10,412	11,323
Pseudo *R*^2^ (McFadden)	0.702	0.705	0.702	0.697	0.716	0.732

Marginal effects in italics, z-statistics in parentheses; *, ** and *** represent statistical significance at 10%, 5% and 1% levels, respectively.

Finally, we explore the differences in frequency of items recalled for the month response task and the year response task, compared with the week response task. We find statistically significant responses for participants in the Utilitarian Activity future fluency category, with responses being on average greater in the future week question than the future month question. We also find a relationship with Temporal Landmark and future fluency, in that respondents on average provide more Temporal Landmark items for the month question as opposed to the week question. When we explore the differences between weekly and yearly responses, we find that all six specifications are statistically significant. Here we see that study participants recall less Utilitarian Activities for the year in advance or the previous week, when compared with a week in advance or previous. Interestingly, for both Discretionary Activities and Temporal Landmarks, participants list more responses in both the past and future fluency times for one year, compared to only one week.

## Discussion

Engaging in anticipatory behaviours towards temporal events, and actively taking part in discretionary activity scheduling, generates utility in the present – regardless of the scale of the temporal activity. We identify that in contexts with reduced agency, individuals increase the salience of otherwise minutia events (micro-moments) and employ anchoring heuristics^43^ for positive anticipatory emotions and temporal manipulation. Research has shown that anticipation gives people some degree of control over an uncertain future, generates immediate hedonic wellbeing, often acting as a self-regulation strategy^7,44^. In this study, through our qualitative categorization of the variable of interest (i.e. temporal events) we extend the discussion about behaviours that support individuals in manipulating subjective perceptions of time (i.e. time work) to also incorporate anticipation. When faced with ‘empty time’, we find participants expending their effort to envisage more accurate construal’s of emotionally laden events in the future (i.e. Temporal Landmarks, Discretionary Activities), thereby manipulating their perceptions of time by reducing the psychological distance to the event in question.

This form of ‘mental time travel’ via engagement in anticipatory processes is particularly beneficial when faced with reduced agency^15^ and should thereby be considered (and encouraged) as a type of time work^9,45^. Our multivariate analysis reinforces this proposition with statistically significant results regarding past and future fluency discretionary activities, demonstrating that under contexts of reduced agency (i.e. stringency index) participants turn to actively scheduling (and thereby recalling) emotionally laden micro-events as mechanisms of wellbeing generation. Connecting the Stringency Index with this type of time work (scheduling discretionary events) demonstrates the role of anticipation as an adaptive strategy in dealing with situations of limited or constrained agency, whereby one’s available timework strategies are restricted by organisational, structural and situational demands^46^, as was the case during the pandemic. Interestingly, we also find a statistically significant relationship between high Subjective Confinement scores (i.e. one’s perceived or subjective measure of the duration of the isolation measure) and reported Temporal Landmarks in both the Past and Future Fluency tasks. This may suggest that the more one feels as though they are socially isolated and lonely, the more they turn to salient events as anchors to generate wellbeing benefits. That is, looking forward in anticipation to a Temporal Landmark provides utility for those that are currently dealing with ‘empty time’, while in turn looking back retrospectively at an emotionally charged Temporal Landmark can provide a modality to extend the positive effects of the foci^10^. Those then without positively valanced Temporal Landmarks, either in the past or scheduled for the future, may naturally experience increased difficulties coping with extended ‘empty’ time. Future research would do well to further explore individual differences (such as socio-demographics and personality characteristics) exact impact on such time-work engagement.

It is important to note that the study’s data set, post coding, has a high frequency of participant responses towards Utilitarian Activities in the short and medium term (i.e. week and month, past and future). A simple explanation comes from the construal level theory of psychological distance, where events are represented at a more concrete level the closer they are, and more abstractly when there is greater psychological distance^45^. That said, it is still important to acknowledge the impact of engaging in time work through increased frequency of utilitarian activities in the short term. While our findings confirm the presence of time work, specifically via the mechanism of scheduling^9^, we identify differences between being just ‘busy’ with routine activities vs. in fact positively *looking forward* to events. This is because the qualitative composition of the events punctuating an individual’s temporal landscape is certainly important for one’s wellbeing. Having discretionary or fun activities undoubtedly provides a different ‘texture of time’, when compared with more menial utilitarian activities^17^ especially in times of reduced agency. Our data visualization in Fig. 1 (corroborated by our negative binomial regression results in Table 6) demonstrates such showing an inverse Past and Future Fluency pattern for weekly vs. monthly recall of Temporal Landmarks vs. Utilitarian Activity frequency. When fewer Temporal Landmarks are reported in the short term (i.e. week), greater numbers of Utilitarian Activities are listed in comparison. The reverse is true when asking participants about activities across the more distant temporal horizon (i.e. a year past or in the future) - a strong indication that Utilitarian Activities become more salient in response to the lack of Temporal Landmarks in focal prominence.

When individuals have limited positive future oriented events (i.e. foci of anticipation), they appear to direct their attention towards actively structuring activities at the micro level, thus modifying their subjective perceptions of time. This relates to the scheduling component of time work^9^, whereby high frequencies of utilitarian activities reflect a desire to keep oneself occupied within the monotony of confinement. Idleness is often something that is dreaded, and therefore people desire a sense of ‘busyness’ as a proxy for meaning or motivation in life under reduced agency^47^. Self-imposed ‘busyness’ (increasing the frequency of utilitarian activities) may then make individuals feel occupied in the short term, but not translate into encoded memory because of the low emotionally intensity or variety of the tasks^48^. Greater routine in life can make experiences less intensive, resulting in the mind retaining them with less clarity or frequency^49^. Research has shown subjective time to accelerate (in memory) as routine and monotony increases^50^. In contrast, greater variety makes a given time period expand in retrospect^51^. Future research would do well to explore this apparent inverse relationship between the salience of Temporal Landmarks and Utilitarian Activities under reduced agency.

From an applied science perspective, our study has identified important sex differences in behaviour, in that males report less discretionary activities across both past and future time points, and more future utilitarian activities as landmarks, compared to their female counterparts. Males have previously been shown to have a more extended future orientation when it comes to economic or occupational areas^52^. Further, while men typically have greater time availability for leisure (compared to women), women have been shown to have more positive leisure experiences by taking greater initiative in scheduling discretionary activities that maximize limited time availability^53^.

Our results also show that women (on average) recall less past Temporal Landmarks compared to men. Such findings correspond with research demonstrating that women tend to have more positive time perspectives (i.e. future-positive time perspective, and expanded-present)^54^. Women are more future oriented than men in their private spheres, thus taking the initiative to schedule Temporal Landmarks that they can anchor to as a way to provide current utility^52^. In contrast, men have a present orientation^55^ and thus may derive more utility in the present from past Temporal Landmarks, suggesting that the power of reminiscence and rosy retrospection can be relatively more important for men (compared to women) within contexts of reduced agency, acting as a way to enhance self-esteem^56^. As previously suggested, further analysis is warranted to connect these sex differentiated behaviours to any specific personality traits, socio-demographic characteristics, or barriers and enablers to engaging in these types of time work within contexts of reduced agency.

### Applications and future directions

Our findings have significant implications for applied science, business and public policy, in establishing a better understanding of the type of Temporal Landmarks and Discretionary Activities that are best suited to influencing an individuals’ perception of time with the hope of positively impacting utility. This deeper understanding can inform the creation of better time management and productivity tools more tailored to the behavioural tendencies and preferences of particular users. Incorporating features that encourage users to better schedule and celebrate Temporal Landmarks (e.g., achievements, deadlines, or leisure activities). Employers can also draw upon the results regarding the impact of emphasizing positively valenced future Temporal Landmark and consider how to structure schedules and milestone frameworks to foster greater motivation and productivity among employees. Testing the effects of, for example, introducing future-focused goal-setting workshops or milestone-driven initiatives upon workplace satisfaction would be an important next step, particularly in highly sex differentiated (e.g. male-dominated) or high-stress industries.

Furthermore, exploring time work in other contexts of reduced agency or empty time (i.e. incarceration and corrective services, hospitals, retirement homes, long haul flights, etc.) would be an obvious and important first step in expanding the impact of this anticipation and time perception for wellbeing. Developing tailored interventions that leverage positively valenced Temporal Landmarks may enhance coping mechanisms, improve mental wellbeing, and foster resilience in individuals with limited agency. For patients in long-term care, rehabilitation or retirement homes, programs could emphasize scheduled activities and future-oriented Temporal Landmarks to create a sense of purpose, improve wellbeing and reduce feelings of stagnation or helplessness^10^. On the other hand, in the context of incarceration and corrective services, scheduling future Temporal Landmarks and Utilitarian Activities for inmates could become an important policy and institutional management tool. Not only reducing inmate idleness and creating a sense of meaningful progression over time^57^, but also generating benefits for prison employees by acting as a mechanism to incentivize or nudge good behaviour and reduce recidivism^58^. Field work (both descriptive and experimental) in differing ecologies would provide unique natural variations, allowing for the exploration of the impact of context dependent shifts or changes in participant agency on the employment of “time work” by individuals and even groups. Providing such novel behavioural data for analysis may in fact challenge current institutional and managerial thinking about best practice and consumer preferences^59^.

By demonstrating the importance of having positively valenced Temporal Landmarks, either in the past or scheduled for the future, our study provides a primer for future research to explore how engaging in such scheduling can be better understood as an adaptive coping mechanism for those within contexts of constraint and reduced agency.

## Electronic supplementary material

Below is the link to the electronic supplementary material.

Supplementary Material 1

## Data Availability

The datasets from the original Blursday study are available in their repository at https://dnacombo.shinyapps.io/Blursday/. The data and code generated or analyzed during the current study are available in our OSF repository 10.17605/OSF.IO/ASJ3U.
