# Supplementary material for: Exploring past and future fluency of temporal landmarks under reduced agency
Source: Sci Rep. 2025 May 7;15:15920. doi: 10.1038/s41598-025-00530-4 (PMC12059124; doi:10.1038/s41598-025-00530-4)
Supplement: Supplementary file 1 — Supplementary Material 1 [file 41598_2025_530_MOESM1_ESM.docx]

**Exploring Past and Future Fluency of Temporal Landmarks Under Reduced Agency**

**Supplementary Information**

**Table S1:** Descriptive statistics – subcategory breakdown of count and percentage for each of the four top-level categories (Temporal Landmarks, Utilitarian Activities, Discretionary Activities, Evaluations).

| **Main Category** | **Label Type** | **Full Subcategory Label** | **Count** | **Percentage** |
| --- | --- | --- | --- | --- |
| Temporal Landmarks | Primary | primary_personal_temporal_landmark | 6138 | 8.783378 |
|  |  | primary_calendar_temporal_landmark | 1483 | 2.122149 |
|  |  | primary_reference_points | 614 | 0.878624 |
|  |  | primary_personal_narrative_events | 1708 | 2.44412 |
|  |  | primary_facts_of_life_experiences | 1240 | 1.77442 |
|  |  | primary_absence_of_activity | 490 | 0.701182 |
|  | Secondary | secondary_personal_temporal_landmark | 549 | 0.78561 |
|  |  | secondary_calendar_temporal_landmark | 149 | 0.213217 |
|  |  | secondary_reference_points | 625 | 0.894365 |
|  |  | secondary_personal_narrative_events | 278 | 0.397813 |
|  |  | secondary_facts_of_life_experiences | 122 | 0.17458 |
|  |  | secondary_absence_of_activity | 63 | 0.090152 |
| Utilitarian Activities | Primary | primary_household_obligations | 4206 | 6.018717 |
|  |  | primary_physiological_needs_personal_care | 1844 | 2.638734 |
|  |  | primary_work_school_activities | 8884 | 12.71286 |
|  |  | primary_services | 2637 | 3.773504 |
|  |  | primary_care_duties | 489 | 0.699751 |
|  |  | primary_return_to_routine | 516 | 0.738388 |
|  |  | primary_career_planning | 1092 | 1.562634 |
|  |  | primary_civic_duties | 153 | 0.21894 |
|  | Secondary | secondary_household_obligations | 317 | 0.453622 |
|  |  | secondary_physiological_needs_personal_care | 82 | 0.117341 |
|  |  | secondary_work_school_activities | 382 | 0.546636 |
|  |  | secondary_services | 124 | 0.177442 |
|  |  | secondary_care_duties | 46 | 0.065825 |
|  |  | secondary_return_to_routine | 79 | 0.113048 |
|  |  | secondary_career_planning | 163 | 0.23325 |
|  |  | secondary_civic_duties | 8 | 0.011448 |
| Discretionary Activities | Primary | primary_recreation | 10821 | 15.48467 |
|  |  | primary_entertainment | 3647 | 5.218797 |
|  |  | primary_social | 5840 | 8.356945 |
|  |  | primary_family | 1961 | 2.806159 |
|  |  | primary_altruistic | 172 | 0.246129 |
|  |  | primary_aspirational | 384 | 0.549498 |
|  |  | primary_recreation_services | 13 | 0.018603 |
|  |  | primary_shopping | 2167 | 3.100942 |
|  |  | primary_introspection | 492 | 0.704044 |
|  |  | primary_home_improvement | 335 | 0.47938 |
|  |  | primary_travel | 3457 | 4.946911 |
|  |  | primary_self_improvement | 782 | 1.119029 |
|  |  | primary_new_connection_seeking | 364 | 0.520878 |
|  |  | primary_spiritual_activities | 113 | 0.161701 |
|  | Secondary | secondary_recreation | 746 | 1.067514 |
|  |  | secondary_entertainment | 88 | 0.125927 |
|  |  | secondary_social | 517 | 0.739819 |
|  |  | secondary_family | 284 | 0.406399 |
|  |  | secondary_altruistic | 18 | 0.025758 |
|  |  | secondary_aspirational | 41 | 0.05867 |
|  |  | secondary_recreation_services | 0 | 0 |
|  |  | secondary_shopping | 82 | 0.117341 |
|  |  | secondary_introspection | 27 | 0.038637 |
|  |  | secondary_home_improvement | 36 | 0.051515 |
|  |  | secondary_travel | 306 | 0.437881 |
|  |  | secondary_self_improvement | 94 | 0.134512 |
|  |  | secondary_new_connection_seeking | 14 | 0.020034 |
|  |  | secondary_spiritual_activities | 12 | 0.017172 |
| Evaluations | Primary | primary_negative | 1782 | 2.550013 |
|  |  | primary_neutral | 608 | 0.870038 |
|  |  | primary_positive | 725 | 1.037463 |
|  |  | primary_lockdown | 334 | 0.477949 |
|  | Secondary | secondary_negative | 496 | 0.709768 |
|  |  | secondary_neutral | 154 | 0.220371 |
|  |  | secondary_positive | 299 | 0.427864 |
|  |  | secondary_lockdown | 82 | 0.117341 |

Table S2: Internal reliability metrics for the three subsets – Humans Only, Lucus (expert) and AI; Sarah, Sean (novices) and AI - across the four top-level categories (Temporal Landmarks, Utilitarian Activities, Discretionary Activities, Evaluations).

| **Category** | **Subset** | **Fleiss' Kappa (All)** | **Cohen's Kappa (All Pairs)** | **% Agreement (All)** | **Fleiss' Kappa (Subset)** | **Cohen's Kappa (Subset Pairs)** | **% Agreement (Subset)** |
| --- | --- | --- | --- | --- | --- | --- | --- |
| Discretionary Activities | Humans Only | 0.9218 | [0.9691, 0.9461, 0. 8851] | 92.57% | 0.9184 | [0.9321, 0.9267] | 94.19% |
|  | Lucas and AI |  |  |  | 0.9276 | [0.6915] | 96.58% |
|  | Sarah, Sean and AI |  |  |  | 0.8700 | [0.9904, 0.7912] | 90.53% |
| Evaluations | Humans Only | 0.7555 | [0.8533, 0.7636, 0.5602] | 97.77% | 0.8670 | [0.9061, 0.7941] | 98.84% |
|  | Lucas and AI |  |  |  | 0.8756 | [0.87558] | 99.15% |
|  | Sarah, Sean and AI |  |  |  | 0.7290 | [0.9475, 0.6082] | 96.84% |
| Temporal Landmarks | Humans Only | 0.7649 | [0.7295, 0.7452, 0.7181] | 91.08% | 0.7438 | [0.7077, 0.7343] | 90.99% |
|  | Lucas and AI |  |  |  | 0.7041 | [0.7041] | 93.45% |
|  | Sarah, Sean and AI |  |  |  | 0.7958 | [1.0, 0.7045] | 91.26% |
| Utilitarian Activities | Humans Only | 0.7703 | [0.8252 0.7493, 0.7470] | 82.16% | 0.7730 | [0.81782, 0.7707] | 86.63% |
|  | Lucas and AI |  |  |  | 0.8140 | [0.8140] | 92.31% |
|  | Sarah, Sean and AI |  |  |  | 0.7672 | [0.9830, 0.6491] | 85.92% |

* *Note:* There were 3819 responses in total from the four coders (3 human RAs, 1 AI). The “expert” human RA coded 1,105 responses, the “AI” coded 795 responses, and the two “novice” human RAs coded 1,141 and 778 responses, respectively.


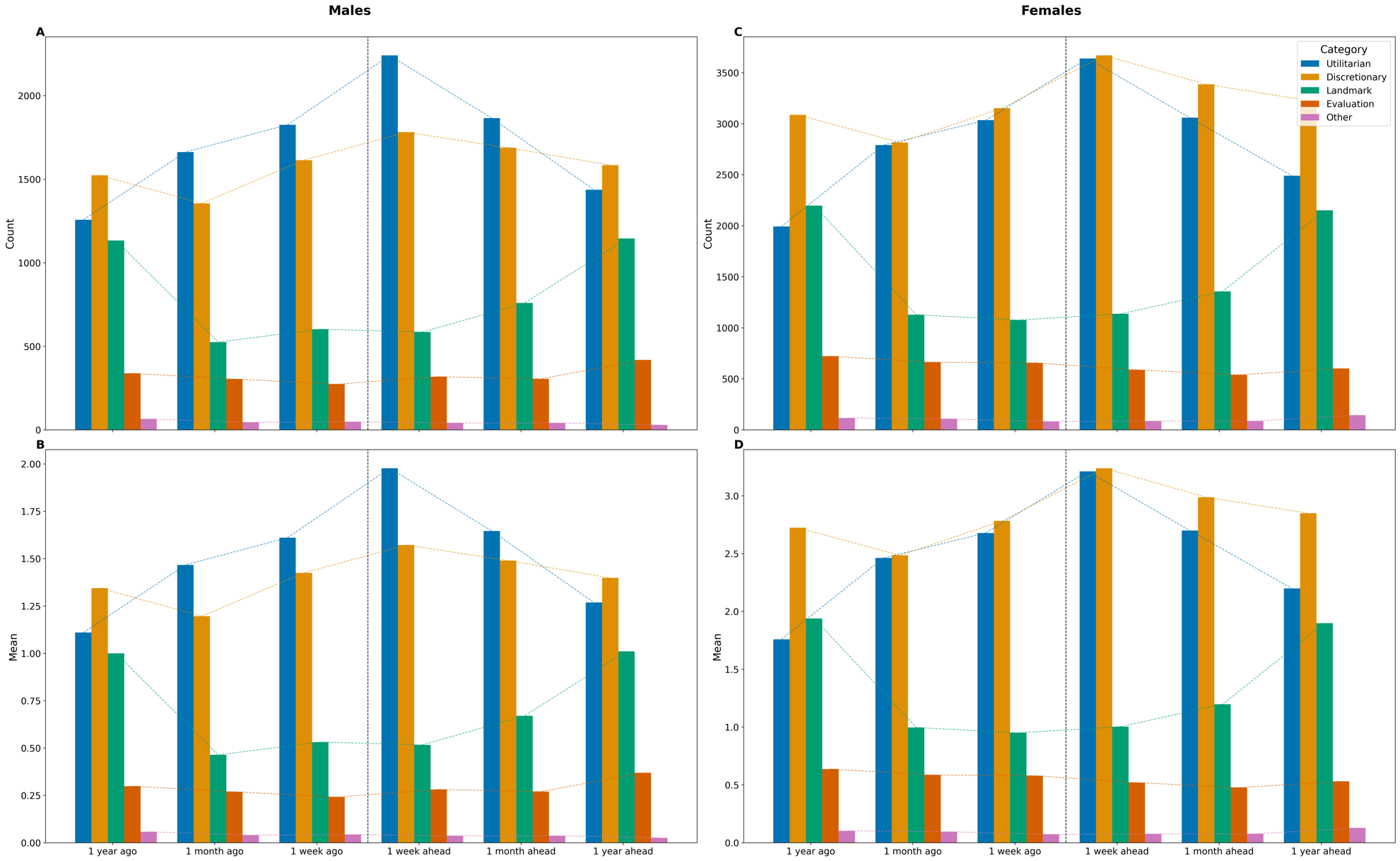


Fig. S1: Same as Fig.1 in the main text but with sex differences, and frequency counts (A, C) per person also.

Table S3: Means difference between Past/Future Fluency for each of the four primary-level activity categories.

| **Variable** | **Past Mean** | **Future Mean** | **Mean Diff.** | **T-Test P-Value** |
| --- | --- | --- | --- | --- |
| *Utilitarian_count* | 4427.333 | 5182.333 | 755.000 | 0.389 |
| *Discretionary_count* | 4827.000 | 5376.667 | 549.667 | 0.116 |
| *Landmark_count* | 2359.667 | 2514.000 | 154.333 | 0.849 |
| *Evaluation_count* | 1047.000 | 965.000 | -82.000 | 0.316 |
| *Other_count* | 164.667 | 149.667 | -15.000 | 0.574 |
| *Utilitarian_mean* | 3.908 | 4.574 | 0.666 | 0.389 |
| *Discretionary_mean* | 4.260 | 4.746 | 0.485 | 0.116 |
| *Lndmark_mean* | 2.083 | 2.219 | 0.136 | 0.849 |
| *Evaluation_mean* | 0.924 | 0.852 | -0.072 | 0.316 |
| *Other_mean* | 0.145 | 0.132 | -0.013 | 0.574 |
| *Utilitarian_count_person* | 3.908 | 4.574 | 0.666 | 0.3889 |
| *Discretionary_count_person* | 4.26 | 4.746 | 0.485 | 0.1164 |
| *Landmark_count_ person* | 2.083 | 2.219 | 0.136 | 0.8493 |
| *Evaluation_count_ person* | 0.924 | 0.852 | -0.072 | 0.3162 |
| *Other_count_ person* | 0.145 | 0.132 | -0.013 | 0.5742 |
| *Utilitarian_count_task* | 0.38 | 0.403 | 0.024 | 0.6695 |
| *Discretionary_count_task* | 0.414 | 0.421 | 0.006 | 0.5005 |
| *Landmark_count_task* | 0.204 | 0.2 | -0.004 | 0.9618 |
| *Evaluation_count_task* | 0.09 | 0.076 | -0.014 | 0.1642 |
| *Other_count_task* | 0.014 | 0.012 | -0.002 | 0.3921 |

*Note:* *** = p < 0.01, ** = p < 0.05, * = p < 0.1.


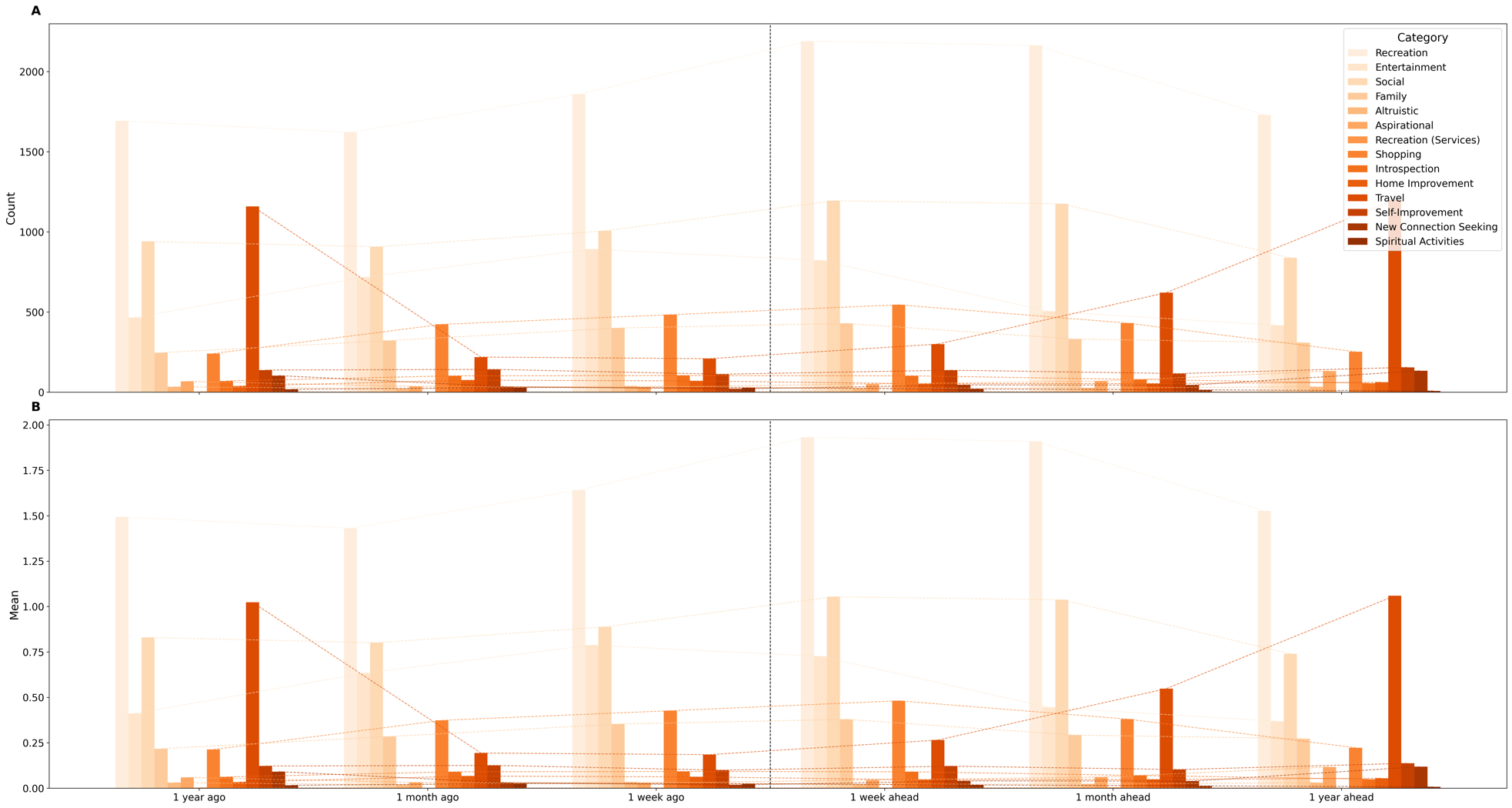


Fig. S2: Same as Fig.1 in the main text but with breakdown for Discretionary activities, and also frequency counts per person at primary category level.


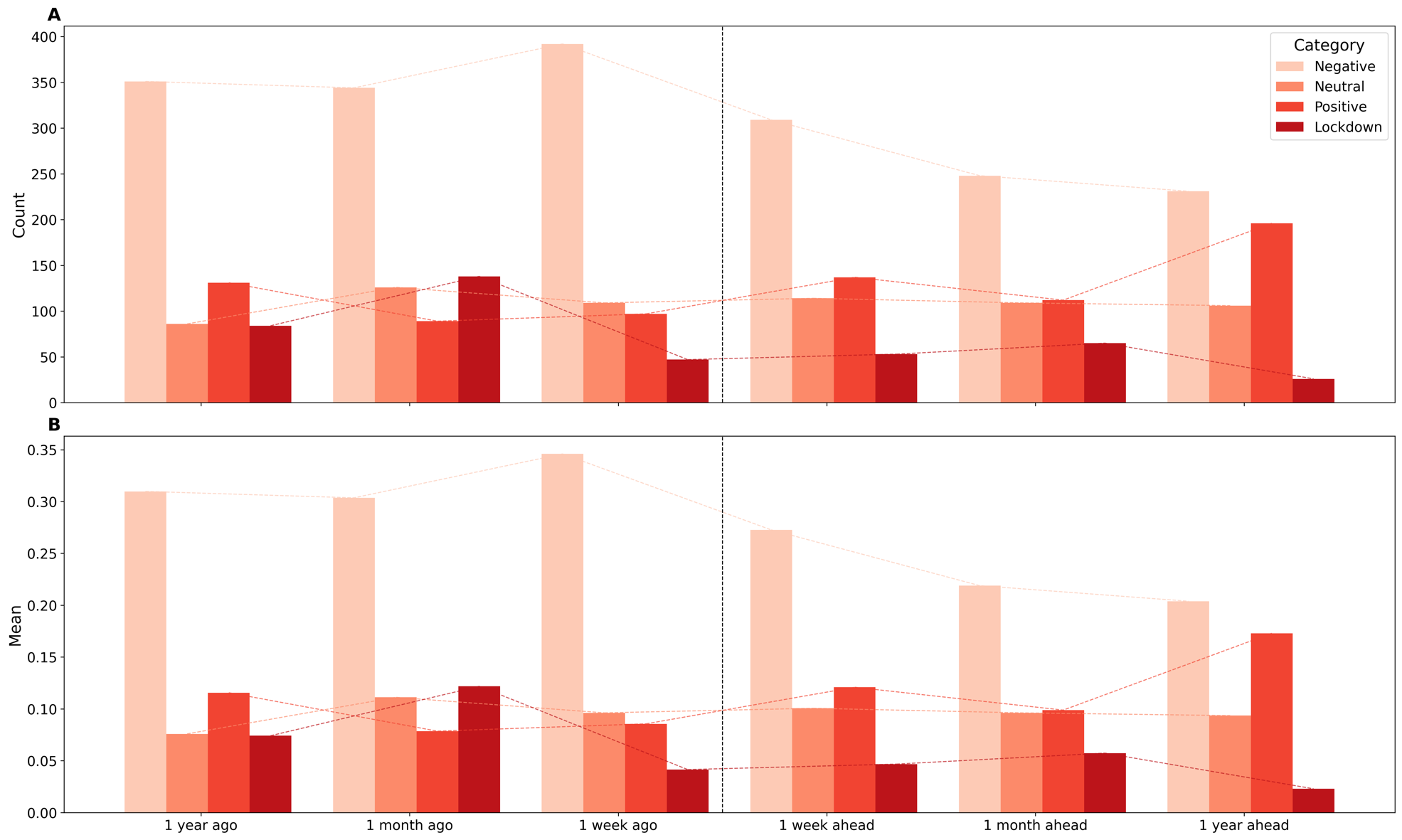


Fig. S3: Same as Fig.1 in the main text but with breakdown for Evaluation activities. Note that A shows frequency count and B shows mean.


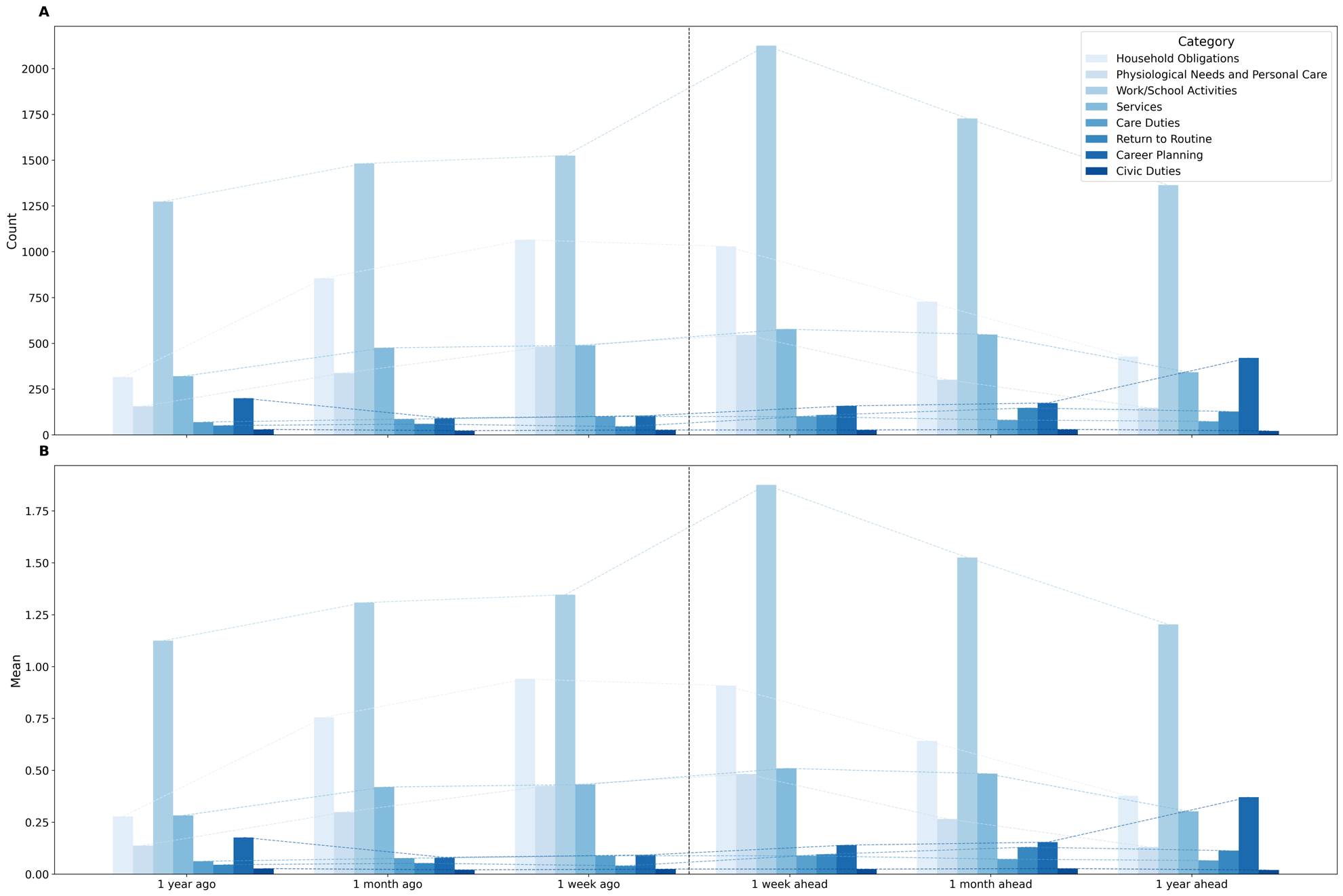


Fig. S4: Same as Fig.1 in the main text but with breakdown for Utilitarian activities. Note that A shows frequency count, and B shows mean.


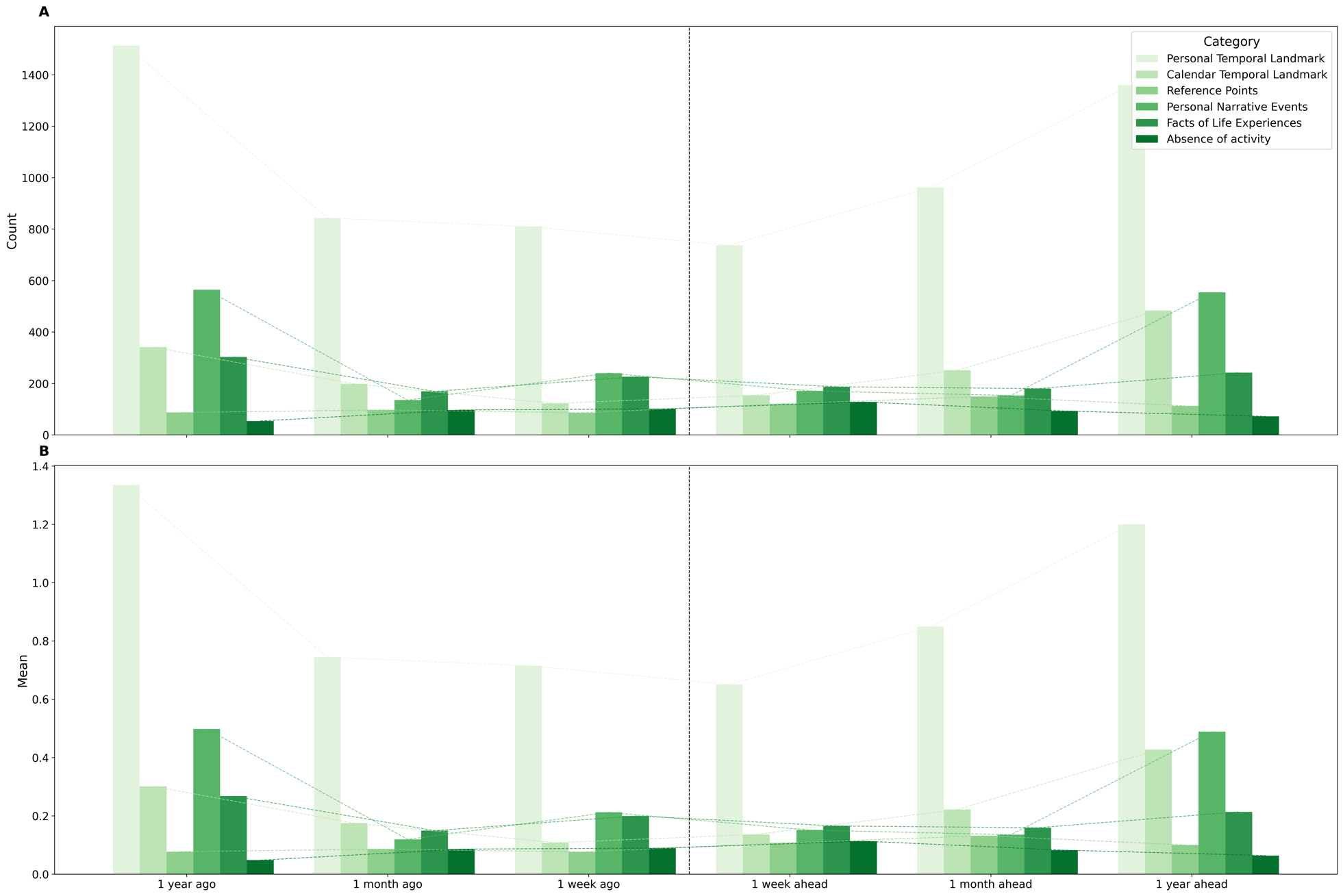


Fig. S5: Same as Fig.1 in the main text but with breakdown for Landmark activities. Note that A shows frequency count, and B shows mean.

Table S4: Regression results (Negative Binomial) across the past/future fluency tasks with demographics and other control variables relating to COVID-19 measurement proxies, including interaction effects.

| Independent Variables | ***Utilitarian Activity*** | | ***Discretionary Activity*** | | ***Temporal Landmark*** | |
| --- | --- | --- | --- | --- | --- | --- |
|  | *Past*  *Fluency* | *Future*  *Fluency* | *Past*  *Fluency* | *Future*  *Fluency* | *Past*  *Fluency* | *Future*  *Fluency* |
| **Age** | -0.001  (-0.594)  *-0.000* | -0.002  (-1.236)  *-0.001* | 0.000 (0.346) *0.000* | 0.002 (1.477) *0.001* | 0.004** (2.061) *0.001* | -0.001  (-0.283)  *-0.000* |
| **Sex**  ***(Male dummy)*** | 0.529* (1.839) *0.145* | -0.053  (-0.198)  *-0.015* | -0.110  (-0.424)  *-0.048* | -0.626*** (-2.597)  *-0.288* | 0.271 (0.726) *0.042* | 1.726*** (4.668) *0.226* |
| **Felt Loneliness** | 0.023* (1.659) *0.006* | 0.013 (1.007) *0.004* | 0.017 (1.513) *0.007* | 0.002 (0.223) *0.001* | -0.036** (-2.121)  *-0.006* | -0.009  (-0.488)  *-0.001* |
| **Reported Loneliness** | -0.014  (-0.910) -*0.004* | -0.013  (-0.935)  *-0.004* | -0.020  (-1.563)  *-0.009* | -0.005  (-0.411)  *-0.002* | 0.046** (2.417) *0.007* | 0.030 (1.516) *0.004* |
| **Stringency Index** | -0.003** (-2.555)  *-0.001* | -0.001  (-0.698)  *-0.000* | 0.001 (1.218) *0.001* | 0.002** (2.334) *0.001* | 0.008*** (4.134) *0.001* | 0.006*** (3.476) *0.001* |
| **Subjective**  **Confinement** | 0.009 (1.609) *0.002* | -0.000  (-0.042)  *-0.000* | -0.002  (-0.527)  *-0.001* | -0.003  (-0.582)  *-0.001* | 0.010 (1.384) *0.002* | 0.021*** (2.949) *0.003* |
| **Confinement**  **Duration** | -0.000  (-1.290)  *-0.000* | 0.000* (1.723) *0.000* | 0.000 (1.573) *0.000* | 0.000 (1.021) *0.000* | 0.000 (0.553) *0.000* | 0.000 (0.462) *0.000* |
| **Screen 2**  ***(One Month)*** | 0.012 (0.216) *0.003* | -0.187*** (-3.376)  *-0.055* | 0.002 (0.030) *0.001* | 0.052 (1.068) *0.024* | 0.003 (0.030) *0.000* | 0.330*** (3.833) *0.043* |
| **Screen 3**  ***(One Year)*** | -0.364*** (-5.817)  *-0.100* | -0.372*** (-6.409)  *-0.109* | 0.159*** (3.181) *0.069* | 0.115** (2.406) *0.053* | 0.616*** (8.236) *0.095* | 0.804*** (10.388) *0.105* |
| **Sex:Felt Loneliness** | 0.006 (0.233) *0.002* | 0.024 (0.990) *0.007* | -0.028  (-1.223)  *-0.012* | -0.010  (-0.484)  *-0.005* | 0.026 (0.796) *0.004* | 0.076** (2.210) *0.010* |
| **Sex:Reported Loneliness** | -0.022  (-0.744)  *-0.006* | -0.028  (-1.033)  *-0.008* | 0.026 (1.031) *0.011* | 0.015 (0.648) *0.007* | -0.017  (-0.475)  *-0.003* | -0.089**  (-2.328)  *-0.012* |
| **Sex:Stringency Index** | -0.002  (-0.930)  *-0.001* | 0.001 (0.618) *0.000* | 0.004** (2.082) *0.002* | 0.006*** (3.352) *0.003* | -0.008*** (-2.776)  *-0.001* | -0.021***  (-7.827)  *-0.003* |
| **Sex:Subjective Confinement** | -0.016*  (-1.688)  *-0.004* | -0.004  (-0.464)  *-0.001* | -0.002  (-0.186)  *-0.001* | 0.005 (0.690) *0.002* | 0.009 (0.732) *0.001* | -0.013  (-1.116)  *-0.002* |
| **Sex:Confinement Duration** | -0.001  (-0.775)  *-0.000* | 0.000 (0.445) *0.000* | -0.002**  (-2.163)  *-0.001* | 0.000 (0.429) *0.000* | 0.003*** (3.614) *0.000* | -0.002**  (-2.082)  *-0.000* |
| **Sex:Screen 2** | 0.048 (0.480) *0.013* | 0.123 (1.298) *0.036* | -0.110  (-1.173)  *-0.048* | -0.022  (-0.250)  *-0.010* | -0.162  (-1.106)  *-0.025* | 0.021 (0.138) *0.003* |
| **Sex:Screen 3** | -0.022  (-0.195)  *-0.006* | 0.018 (0.177) *0.005* | -0.193**  (-2.118)  *-0.083* | -0.063  (-0.729)  *-0.029* | -0.032  (-0.251)  *-0.005* | -0.082  (-0.592)  *-0.011* |
| **Intercept** | -1.142*** (0.000) *0.000* | -1.044*** (0.000) *0.000* | -0.999*** (0.000) *0.000* | -1.025*** (0.000) *0.000* | -2.900*** (0.000) *0.000* | -3.227*** (0.000) *0.000* |
| ***N*** | 10412 | 11323 | 10412 | 11323 | 10412 | 11323 |
| **Pseudo *R^2^* (McFadden)** | 0.703 | 0.706 | 0.702 | 0.697 | 0.717 | 0.734 |

*Notes:* Marginal effects in italics, z-statistics in parentheses; *, ** and *** represent statistical significance at 10%, 5% and 1% levels, respectively.
